# Lipidome Analysis of Oropharyngeal Tumor Tissues Using Nanosecond Infrared Laser (NIRL) Tissue Sampling and Subsequent Mass Spectrometry

**DOI:** 10.3390/ijms24097820

**Published:** 2023-04-25

**Authors:** Rupert Stadlhofer, Manuela Moritz, Marceline M. Fuh, Jörg Heeren, Henrike Zech, Till S. Clauditz, Hartmut Schlüter, Christian S. Betz, Dennis Eggert, Arne Böttcher, Jan Hahn

**Affiliations:** 1 Department of Otorhinolaryngology, University Medical Center Hamburg-Eppendorf, Martinistraße 52, 20246 Hamburg, Germany; 2 Section/Core Facility Mass Spectrometric Proteomics, Diagnostic Center, University Medical Center Hamburg-Eppendorf, Martinistraße 52, 20246 Hamburg, Germany; ma.moritz@uke.de (M.M.); ja.hahn@uke.de (J.H.); 3 Department of Biochemistry and Molecular Cell Biology, Center for Experimental Medicine, University Medical Center Hamburg-Eppendorf, Martinistraße 52, 20246 Hamburg, Germany; 4 Mildred Scheel Cancer Career Center HaTriCS4, University Medical Center Hamburg-Eppendorf, Martinistraße 52, 20246 Hamburg, Germany; 5 Department of Pathology, Diagnostic Center, University Medical Center Hamburg-Eppendorf, Martinistraße 52, 20246 Hamburg, Germany

**Keywords:** nanosecond infrared laser, laser ablation, mass spectrometry, lipidomics, HNSCC, OPSCC, HPV

## Abstract

Ultrashort pulse infrared lasers can simultaneously sample and homogenize biological tissue using desorption by impulsive vibrational excitation (DIVE). With growing attention on alterations in lipid metabolism in malignant disease, mass spectrometry (MS)-based lipidomic analysis has become an emerging topic in cancer research. In this pilot study, we investigated the feasibility of tissue sampling with a nanosecond infrared laser (NIRL) for the subsequent lipidomic analysis of oropharyngeal tissues, and its potential to discriminate oropharyngeal squamous cell carcinoma (OPSCC) from non-tumorous oropharyngeal tissue. Eleven fresh frozen oropharyngeal tissue samples were ablated. The produced aerosols were collected by a glass fiber filter, and the lipidomes were analyzed with mass spectrometry. Data was evaluated by principal component analysis and Welch’s *t*-tests. Lipid profiles comprised 13 lipid classes and up to 755 lipid species. We found significant inter- and intrapatient alterations in lipid profiles for tumor and non-tumor samples (*p*-value < 0.05, two-fold difference). Thus, NIRL tissue sampling with consecutive MS lipidomic analysis is a feasible and promising approach for the differentiation of OPSCC and non-tumorous oropharyngeal tissue and may provide new insights into lipid composition alterations in OPSCC.

## 1. Introduction

Oropharyngeal squamous cell carcinomas (OPSCCs) are a subgroup of head and neck squamous cell carcinomas (HNSCCs) and were estimated to be responsible for 98,412 new cases (0.5% of total cancer incidence) and 48,143 deaths (0.5% of total cancer morbidity) worldwide in 2020 [1]. The current increase in human papillomavirus (HPV)-related OPSCC has countered the positive effects of global efforts to reduce other risk factors such as tobacco usage and alcohol, thus leading to rising incidence overall [2,3]. Compared to the traditional risk factors, HPV-related OPSCC is more prevalent in younger patients and exhibits different biological behavior that tends to be less aggressive and have a better response to treatment [4]. Despite the ongoing development of new treatment strategies, the therapeutic handling of these malignancies remains challenging and requires a multidisciplinary approach [5,6].

Significant progress has been made in understanding the impact of lipid metabolism on cancer formation and progression. The dysregulation of lipid metabolism is known to contribute to the pathogenesis and progression of cancer. Alterations in lipid metabolism, including synthesis, uptake and storage, have been shown to support the growth, survival, and proliferation of cancer cells, as well as their ability to invade and metastasize. Therefore, the analysis of the lipid composition of tumor cells can provide insights into these mechanisms, thus indicating the high potential value of lipid-based molecular analysis of malignant diseases [7,8,9,10,11]. For this, several studies have also been focused on the lipidome analysis of HNSCCs to investigate alterations in lipid metabolism and identify potential biomarkers. However, HNSCCs originate from different anatomical regions such as the oral cavity, larynx, and pharynx. While lipidome analyses of SCCs of the oral cavity and larynx have been performed in previous studies [12,13,14], there is still a lack of knowledge about the lipidome for squamous cell carcinomas of the oropharynx (OPSCCs), thus highlighting the necessity of our study.

Mass spectrometry (MS)-based approaches are an essential tool for gaining comprehensive insights into cellular lipid profiles and their pathologic alterations [15,16]. Furthermore, adequate surgical removal of the tumor and, especially, the status of the surgical margin are important factors for individual outcomes in HNSCC, especially in terms of prognosis, adjuvant treatment, and quality of life [17,18]. In oncologic surgery, the differentiation of preservable healthy tissue versus diseased tissue that needs to be resected is usually performed intraoperatively. Even though the surgeon evaluates the tumor margins using preoperative imaging (size, shape, [micro]environment) there is a dependence on intraoperative tactile and visual information that is combined with the surgeon’s experience [19,20,21,22]. The gold standard for confirming sufficient resection margins is an intraoperative pathologist consultation with frozen sections [17,23,24]. The overall adequacy of using intraoperative frozen sections is >95% in HNSCC cases, but there are several downsides to this technique [25]. In addition to being a time-consuming procedure with interpretation and sampling errors, there are also challenges associated with close margin situations (<5 mm) that can cause sensitivity to drop below 40% for resections of head and neck tumors, thereby leading to positive margins becoming apparent on postoperative histopathologic examination of the complete tumor specimen [23,26,27,28,29].

In the last two decades, great efforts have been made to improve surgical guidance by enhancing tissue characterization on a molecular level through MS-based approaches. In these techniques, MS is used to analyze the molecular information of a tissue sample and, in particular, to identify tissue-specific alterations in lipids and proteins. However, the techniques vary in the way the samples are obtained. The application of an electrosurgical knife (iKnife) [30], desorption electrospray ionization (DESI) [31,32], and liquid extraction surface analysis [33] have all been suggested as potential methods.

Ultrashort pulse mid-infrared lasers (IRL) with pulse widths in the picosecond or nanosecond regime are an alternative emerging technology for tissue sampling, whereby the ablation occurs via desorption by impulsive excitation (DIVE) [12,34,35,36]. The specific wavelength of 2940 nm targets O-H bonds in the tissue’s water molecules. The energy from the laser is almost completely converted into translational energy caused by the vibrational motion of the symmetric O-H stretch band, rather than being transferred to the surrounding tissue by thermal or acoustic transport. The water molecules are driven into the gas phase in a much shorter time scale, thereby decomposing the irradiated tissue and preventing significant collateral damage in the neighboring structures [37,38,39]. Besides the resulting high cutting precision and significant superiority concerning wound healing and scar formation compared with other lasers, the application of DIVE generates an aerosol that is particularly suitable for subsequent differential quantitative proteomics and lipidomics. Several studies have tested the function of real-time MS concepts for tissue differentiation [32,40,41]. Utilizing laser-based aerosolization [12,42,43] or tissue surface extraction [33] combined with real-time MS enables the characterization of different tissue pathologies (cancer, infection, or inflammation) within seconds based on lipid profile signatures. However, to classify the molecular profile of different tissue types, spectral libraries must be created, and the collected data has to be validated across a large population due to significant molecular heterogeneities in human cancer.

In a recent study, we demonstrated tissue sampling and homogenization using a nanosecond infrared laser (NIRL) for MS proteomics with high spatial resolution [44,45]. Here, we translate this knowledge to the field of head and neck tumors.

In this pilot study, we combined NIRL ablation-based tissue sampling with a shotgun lipidomics approach. By using lipid extraction prior to mass spectrometric measurement and differential mobility separation for targeted lipid analysis, isobaric molecular species can be separated and quantified. With this in-depth lipidome analysis, we achieved the quantification of 755 lipid species across 13 lipid classes, which enabled us to analyze the lipid profiles of OPSCCs and compare them to non-tumorous oropharyngeal tissue samples.

## 2. Results

In total, 11 oropharyngeal tissue samples from four patients with HPV-positive OPSCC were sampled by NIRL ablation. For differential quantitative lipidome analysis, OPSCC samples and non-tumorous oropharyngeal tissue from the adjacent areas were taken from each patient (*n* = 7 OPSCC and *n* = 4 healthy mucosa samples). Three technical replicates were obtained from each of the OPSCC and adjacent healthy tissue samples. The demographic and clinical data for all patients are presented in Table 1, and the individual demographic and clinical patient data are presented in Table 2. Table 3 shows the correlation between the sample labels and the individual patients.

**Figure 1:**
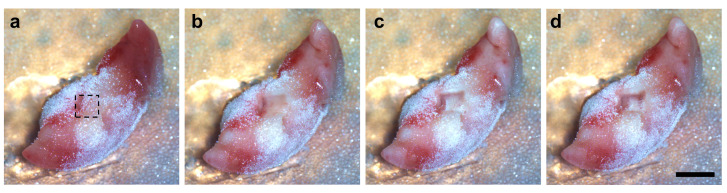
Images of the ablation process on the resected and frozen tissue of sample B-1 placed on the target of the sampling instrument. Images taken before ablation (**a**) and after each of the three replicate ablations, which were performed in a layer-wise fashion (**b**–**d**). The dashed line indicates the edges of the ablation area. NIRL produced a smooth removal of the tissue without any visible burn marks. Scale bar: 2 mm.

After each ablation, photographs were taken from the ablation site to document the tissue composition inside the sample (Figure 1). The total ablation time was 1 min 35 s. Figure 1b–d show the gentle vaporization of the NIRL ablation without visible burn marks on the tissue samples.

**Figure 2:**
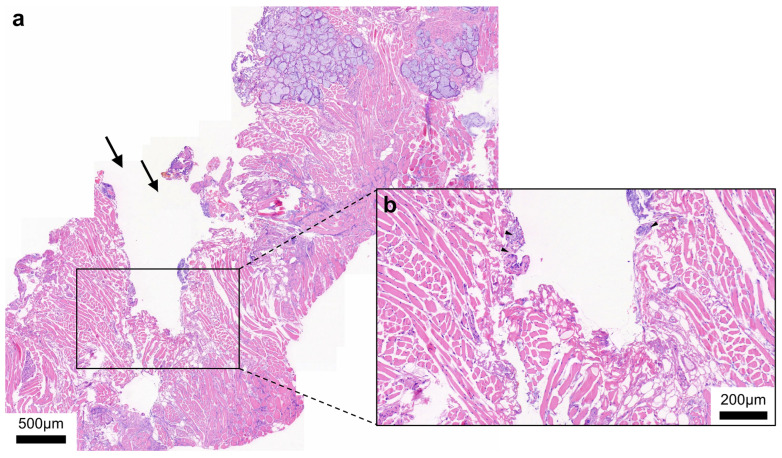
(**a**) Overview of muscle and connective tissue (incision indicated by arrows). (**b**) Higher magnification of the incision site.

Figure 2 shows representative histopathologic H&E stains of tissue sample slides after ablation. It is noteworthy that the NIRL ablation has a high degree of precision.

### 2.1. Distribution of the Identified Lipid Classes

We were able to identify 13 lipid classes and 755 lipid species using mass-spectrometry-based shotgun lipidomics (Supplementary Table S1a,c). The following 13 lipid classes were quantitatively assessed: cholesterol ester (CE); ceramides (CER); diacylglycerides (DAG); dihydroceramides (DCER); free fatty acids (FFA); hexosylceramides (HCER); lysophosphatidylcholine (LCER); lysophosphatidylcholine (LPC); lysophosphatidylethanolamine (LPE); phosphatidylcholine (PC); phosphatidylethanolamine (PE); sphingomyelin (SM); and triacyltriglycerides (TAG).

**Figure 3:**
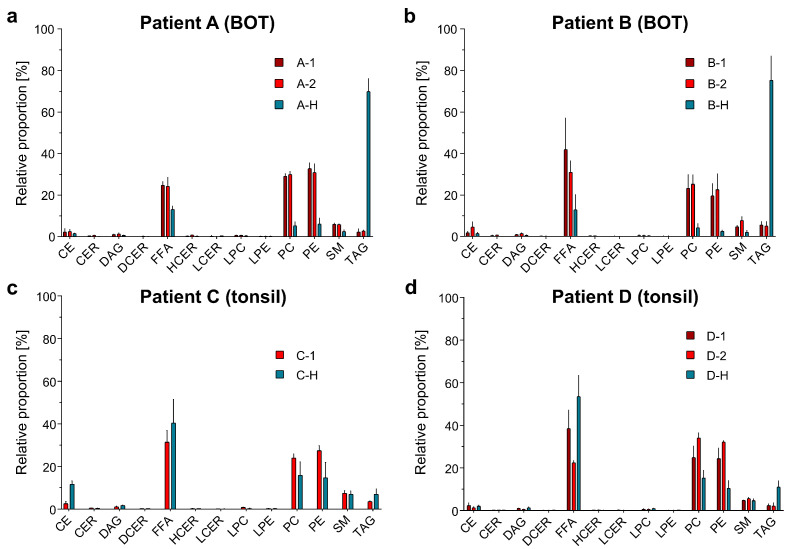
Lipid class composition for all patient samples A–D comparing the OPSCC and non-tumorous oropharyngeal tissue samples (Supplementary Table S1d). Table 3 gives an overview of the samples and their abbreviations. Error bars represent the standard deviation calculated from the three technical replicates per sample. CE: cholesterol esther; CER: ceramide; DAG: diacylglycerol; DCER: dihydroceramide; FFA: free fatty acids; HCER: hexosylceramide; LCER: lactosylceramide; LPC: lysophosphatidylcholine; LPE: lysophosphatidylethanolamine; PC: phosphatidylcholine; PE: phosphatidylethanolamine; SM: sphingomyelin; TAG: triacylglycerol.

Figure 3 shows the lipid compositions (across the 13 lipid classes) for the tumor and adjacent healthy tissue samples from patients A–D. For patient A (Figure 3a), there was an increase in the proportion of FFA, PC, PE, and SM in the OPSCC (base of the tongue; BOT) samples relative to the healthy tissue but a decrease in the proportion of TAG. Similar trends were observed for patient B (Figure 3b). Furthermore, the BOT samples from patients A and B both showed slight increases in the proportions of CE. Although the overall pattern of increases/decreases was similar, the BOT samples from patient A and patient B had different relative proportions of PC, PE, and FFA. The samples from patient A had a higher percentage of PC and PE and a lower percentage of FFA compared with the samples from patient B. For the non-tumorous oropharyngeal tissue samples, the relative proportions for each lipid class were similar for patients A and B (Supplementary Table S1d).

The lipid compositions of the samples taken from tonsillar OPSCCs (patient C and D) are shown in Figure 3c,d. The trends in lipid composition of the OPSCC (tonsil) and adjacent healthy tissue samples differed in some aspects from the trends in the BOT samples. Unlike the BOT samples from patients A and B, which showed an increase in the proportion of FFA, the tonsillar samples from patients C and D showed a decrease in the proportion of FFA. The OPSCC sample from patient C also showed a decrease in the proportion of CE, unlike patients A and B. However, similar to the patients A and B, patients C and D both showed an increase in the proportion of PC and PE in the OPSCC sample compared with the healthy tissue.

Overall, there was an increase in the proportion of PE and PC in OPSCC samples regardless of the tumor location. However, a characteristic specific to the BOT samples was a significant decrease in the proportion of TAG, from about 70% in the non-tumorous oropharyngeal tissue to 5% in the OPSCC samples.

**Figure 4:**
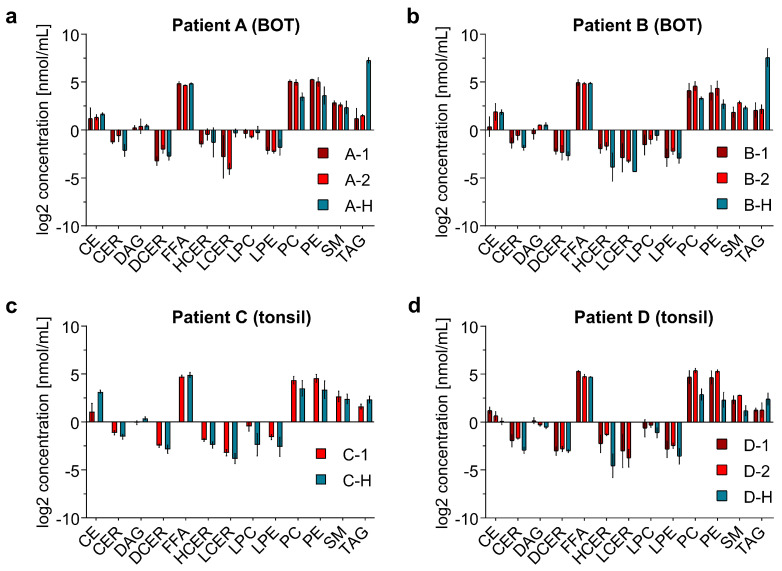
Visualization of log2 concentration for each of the 13 lipid classes quantified in patient samples A–D (Supplementary Table S1e). Table 3 gives an overview of the samples and their abbreviations. Error bars represent the standard deviation calculated from the three technical replicates per sample. CE: cholesterol esther; CER: ceramide; DAG: diacylglycerol; DCER: dihydroceramide; FFA: free fatty acids; HCER: hexosylceramide; LCER: lactosylceramide; LPC: lysophosphatidylcholine; LPE: lysophosphatidylethanolamine; PC: phosphatidylcholine; PE: phosphatidylethanolamine; SM: sphingomyelin; TAG: triacylglycerol.

Figure 4 presents the log2 concentrations of the 13 lipid classes for OPSCC samples and the adjacent healthy tissue samples from each patient. The small standard deviation (calculated from the technical replicates) indicates that the method has good reproducibility. To further evaluate the reproducibility of NIRL-based sampling, Pearson correlation coefficients were calculated (Supplementary Table S1f). The results indicated a higher correlation within technical replicates of the same biological sample compared to different tissue samples. Additionally, all technical replicates within patient samples showed high correlation coefficients above 0.9, therebyindicating the reliability of NIRL-based tissue sampling. The bar graphs show the same pattern for all four patients: The highest concentrations are given for the lipid classes FFA, PC and PE, whereas CER, DCER, HCER, LCER, LPC, and LPE were quantified with very low concentrations of <1 nmol/mL.

In the non-tumorous samples from patients A and B, we measured an exceptionally high concentration of TAG, which even exceeded the log2 concentration of FFA, PC, and PE. This result is consistent with the lipid composition results described above, where a very high proportion of TAG was found to be characteristic of the healthy BOT samples. However, for all patient samples, the TAG concentration was lower in the OPSCC samples than in the healthy samples. Our findings show that, for all patients, the PC and PE lipid concentrations were higher in the OPSCC samples than in the respective non-tumorous oropharyngeal tissue.

### 2.2. Differentiation between Healthy Tissue and Tumor Tissue

**Figure 5:**
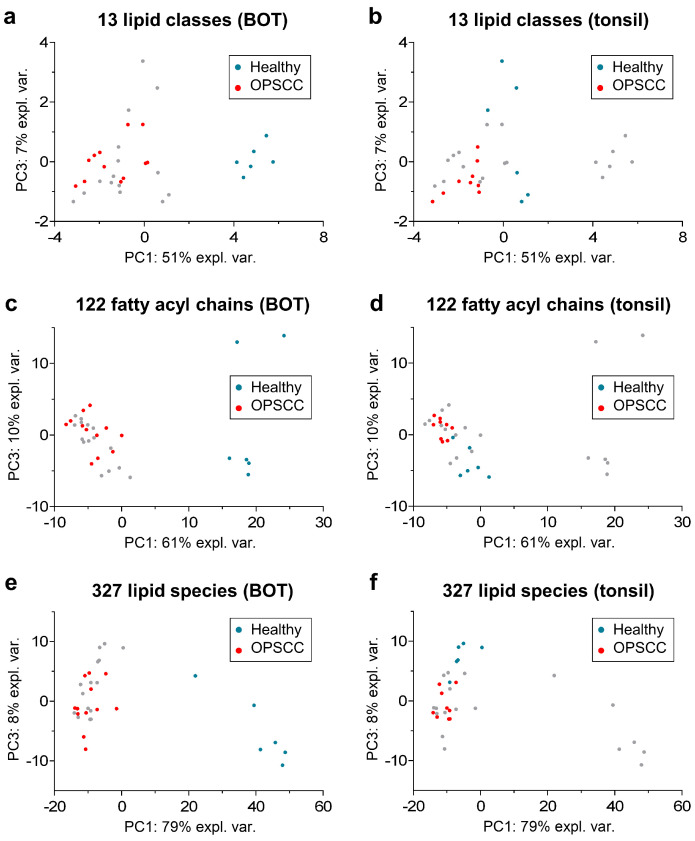
Scatter plot visualization of PCA results calculated with a nonlinear iterative partial least squares (NIPALS) algorithm based on 13 lipid classes, 122 lipid species with same fatty acyl chain length and extent of unsaturation, and 327 individual lipid species (Supplementary Table S2). Each dot represents a single technical replicate, and all technical replicates from all tissue samples are shown in each panel. The healthy (**blue**) and OPSCC (**orange**) replicates corresponding to the panel title (BOT or tonsil) are highlighted in each panel. The other oropharyngeal tissue replicates are shown in gray.

To verify whether the tumor tissue could be clearly distinguished from the adjacent healthy tissue on the basis of the quantified concentrations of the 13 lipid classes, 122 lipid species (summarized by same fatty acyl chain length and degree of unsaturation), and 327 individual lipid species, we performed nonlinear iterative partial least squares (NIPALS) principal component analysis (PCA). Scatter plot visualizations of the PCA results are depicted in Figure 5, with samples from OPSCC and adjacent healthy tissue highlighted for BOT and tonsil samples separately.

For the BOT samples (from patients A and B), there was a clear differentiation of tumorous and healthy tissue along the PC1, regardless of whether the NIPALS PCA was based on quantified lipid classes, lipid species with the same fatty acyl chain length and extent of unsaturation, or individual lipid species (Figure 5a,c,e). For the tonsil samples, OPSCC and healthy tissue samples were also separated along the PC1 (Figure 5b,d). Based on both the lipid species summarized by their fatty acyl chain and individual lipid species, the different sample types could also be separated on the basis of PC3 (Figure 5d,f). Furthermore, the OPSCC samples for the tonsil and BOT were clustered in a very similar area in the scatterplot visualizations, but the non-tumorous samples for the tonsil and BOT were in different parts of vector space. From this, it can be assumed that there are similarities in the lipid profiles of the OPSCC samples from different tumor locations but that non-tumorous samples from the BOT and tonsil have different lipid profiles. This is consistent with the different TAG concentrations in the non-tumor tissues from the BOT and tonsil (Figure 4).

**Figure 6:**
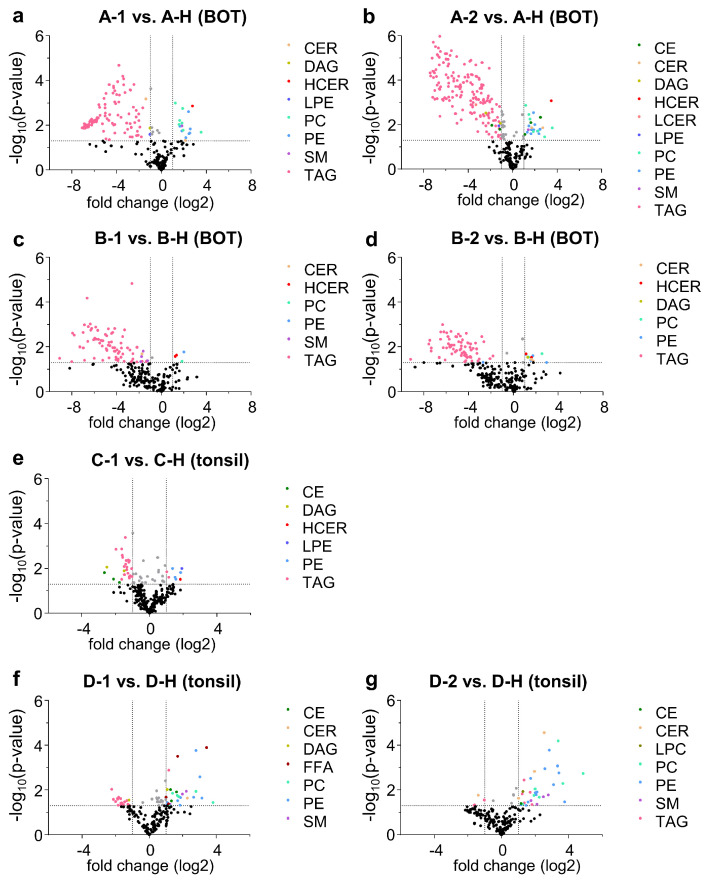
Volcano plots showing the results of the Welch’s tests. The lipid species with a significant difference (*p*-value ≤ 0.05, two-fold change) in their concentrations between the indicated samples are highlighted. Table 3 gives an overview of the samples and their abbreviations. Lipid species with a *p*-value > 0.05 are shown in black, and those with a *p*-value ≤ 0.05 but a fold change < 2 are shown in gray. CE: cholesterol esther; CER: ceramides; DAG: diacylglycerol; DCER: dihydroceramides; FFA: free fatty acids; HCER: hexosylceramides; LCER: lactosylceramides; LPC: lysophosphatidylcholine; LPE: lysophosphatidylethanolamine; PC: phosphatidylcholine; PE: phosphatidylethanolamine; SM: sphingomyelin; TAG: triacylglycerol.

Welch’s test was used to identify significant differences between the lipidomes of the OPSCC and healthy samples for each patient. As differences in the biological replicates are already shown in Figure 4 and Figure 5, Welch’s test was performed for each biological replicate, including three corresponding technical replicates. Figure 6 shows the results visualized as volcano plots. Significant two-fold changes (*p* < 0.05) for lipid classes are highlighted.

For samples that originated from the BOT (from patients A and B; Figure 6a–d) there was a very strong deflection of the TAG. From the log2 fold change, the concentration was 60-fold higher in the healthy tissue than in the OPSCC tissue. By contrast, the lipid classes PE, PC, and HCER showed a significant shift to higher concentrations in the OPSCC tissue compared with the non-tumorous oropharyngeal tissue.

We observed a significantly higher concentration of PC and PE in the OPSCC tissue from patient D (Figure 6f,g) compared with the corresponding healthy tissue: the difference was up to 32-fold, which was much higher than the difference in PC for the BOT samples (2–4-fold change). Another interesting lipid class was CE, which showed a significant difference in concentration between OPSCC and healthy tissue from patients A, C, and D. There was an increase in CE lipid species in OPSCC tissue from patients A and D but a decrease in OPSCC tissue from patient C.

Overall, when comparing the BOT and tonsil samples, the tonsil samples had fewer lipid species with significantly lower concentrations in the OPSCC samples than the healthy samples. This is highlighted in Table 4, which displays the number of lipid species with a significant difference (higher or lower) between OPSCC and healthy tissue, according to the results of the Welch’s tests.

All sample pairs showed some significant differences; however, there were a greater number of significant differences for the sample pairs obtained from the BOT than from the tonsil.

## 3. Discussion

In this pilot study, we successfully demonstrated the feasibility of differentiating oropharyngeal tissue types using an innovative NIRL ablation setup with subsequent MS-based shotgun lipidomic analysis. This study shows the potential of the IRL-based platform to analyze and discriminate fresh frozen tissue samples from tumor (OPSCC) and non-tumor regions in the oropharynx and revealed significantly different lipid class compositions in healthy and cancerous tissue obtained from patients with differing gender, age, and habits (including alcohol and tobacco consumption).

We focused in particular on the exact determination of the lipid composition of the analyzed tissue to provide valuable information for our collective understanding of the alterations in lipid composition in OPSCCs. Therefore, we chose to use fresh frozen tissue samples rather than formalin-fixed paraffin-embedded (FFPE) samples, as some lipids are not preserved during FFPE processing [46,47,48]. Across all samples, we were able to identify 13 lipid classes with up to 755 individual lipid species.

In the second step, we analyzed the specific lipid classes and species for tissue discriminability. We found significant changes in the concentrations of lipid classes when comparing tumorous and non-tumorous tissue samples from all given patients. The TAG, CER, PC, and PE lipid classes were the decisive classes for the discrimination of tumorous and non-tumorous samples in our analysis. In particular, the 60-fold decrease of TAG in tumorous samples from the base of the tongue and the 2–4-fold decrease of TAG in some of the tonsillar OPSCC samples make this lipid class potentially important and worth investigating in a larger patient population.

It is noteworthy that previous studies on lipidome changes of other neoplastic entities do not follow a general principal pattern for tumor genesis or progression. Li et al. were able to discriminate prostatic tumor samples from non-tumor samples via changes in CE, CER, nonesterified fatty acids, and TAG [49]. Another study on gastric cancer found relevant changes in the levels of lysophospholipids, PC, PE, PI, phosphoserines, SM, CER, and TAG when comparing cancerous and noncancerous tissue samples [50]. We assume that the patterns of change in lipid classes in our study are also unique to OPSCC. Nevertheless, recently published data by Ogrinc et al. on oral tongue SCC and non-tumor regions showed discrimination based on PC and PE in the positive ion mode and on phosphatidic acid, PI, and phosphatidylserines in the negative ion mode [12].

Similar to the study by Ogrinc et al., we were also confronted with high interpatient variability in lipidomic profiles [12]. Even though we observed a significant decrease in the concentration of TAG in all tumor tissue samples from the base of the tongue, this decrease was not observed in all tonsillar tumors. The same was evident for the increase in the PC and PE in all tumor samples. This may be a result of the macroscopic determination of the material being analyzed. Despite the subsequent histopathological assignment of the residual material, there is a chance that tumor-associated tissue types were present in the ablated region with a resulting alteration in lipid profiles. The presence of the tumor microenvironment is an inevitable factor when studying human specimens: it leads to higher complexity and therefore greater heterogeneity in the examined tissue than when cell line models, xenografts, or organoids are used [51]. Furthermore, the intrapatient heterogeneities found in our patient collective may also be attributed to individual alterations in cancer cell metabolism due to nutrition, lifestyle (e.g., tobacco use), and genetic factors. Despite the small patient cohort (*n* = 4), our findings show the method’s potential to differentiate between OPSCC and non-tumorous oropharyngeal tissues based on significant alterations in lipid profiles. However, the above-mentioned (epi)genetic factors should be considered for future studies in a larger patient cohort, as the lipidome analysis of OPSCCs and the understanding of these factors and their influence on individual lipidomic profiles remains limited [52].

Overall, this study demonstrates the feasibility of lipidomic analysis of oropharyngeal tissue using a NIRL-based ablation setup, which, in principal, allows three-dimensional tissue sampling. The ablated tissue volume needed for the quantitative analysis of lipid classes with the Lipidyzer^TM^ Platform is high when compared with approaches that use proteomics [45,53,54,55]. However, in this study, we successfully analyzed sample volumes of less than 500 nL, which corresponded to a voxel of 800 µm edge length. To our knowledge, this is the smallest reported sample volume for shotgun lipidomics with IRL-based tissue sampling, thus demonstrating the enormous potential of spatial lipidomics. The approach is much faster than classical mechanical homogenization and more volume efficient than the IRL-based sampling described in our previous study, which had an ablation volume of 1 mL (a factor of 2000) and utilized an aerosol cryotrap [40].

Improvements in the efficiency of aerosol capture and post-ablation sample processing, and modification of the MS instrument may help exploit this potential and further improve the spatial resolution of tissue sampling and differentiation. With a further increase in resolution, it may be possible to resolve the heterogeneity of the tumor inside the samples.

This study shows that IRL-based sampling in general—and NIRL-based sampling in particular—combined with tissue homogenization and an aerosol collection system is a very promising concept for future biopsies, as also demonstrated in our previous work [56]. When implemented in an image-guided handheld device or integrated into existing laryngoscopes, this approach could be applied during head and neck cancer screening, disease monitoring, or follow-up examinations. A further potential usage of NIRL MS-based lipidomic tissue sampling is intraoperatively in oncologic surgery, to enable a faster but nevertheless reliable determination of surgical margins in OPSCC. By providing a deeper insight into the lipidome of OPSCCs, this study lays the foundation for such promising approaches.

## 4. Methods

### 4.1. Samples

A total of 11 oropharyngeal tissue samples from four patients were included in this study. The tissue samples were collected from July 2020 to December 2020 under general anesthesia at our institution. Following collection, the tissue samples were immediately rinsed with sodium chloride solution (NaCl 0.9%), transferred to 15 mL centrifuge tubes and frozen in liquid nitrogen. Samples were then stored at –80 °C until further processing. The samples were collected and processed in accordance with the World Medical Association Declaration of Helsinki and the guidelines for experimentation with humans by the Chambers of Physicians of the State of Hamburg. All patients gave written informed consent for their excised tissue to be used for research purposes. The Hamburg Commissioner for Data Protection and Freedom of Information (HmbBfDI) was notified of the collection of head and neck tumor tissue in the context of a biobank, in accordance with local laws (§12 HmbKHG) and the local ethics committee (Ethics commission Hamburg WF-049/09). For histopathologic reconfirmation, representative parts of the tissue samples were H&E stained following the standard operating procedures of the Institute of Pathology. The stained slides were evaluated blind by an expert pathologist to confirm the histological diagnosis.

### 4.2. Ablation Setup

**Figure 7:**
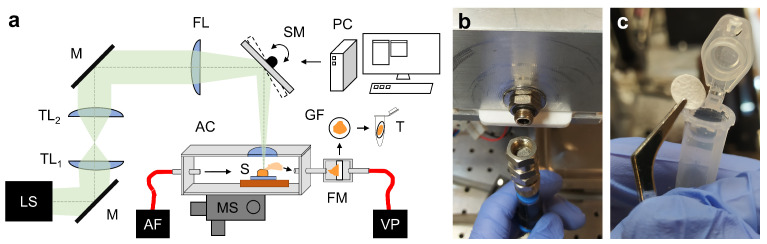
(**a**) Depiction of the ablation setup. (**b**) Filter mount (open). (**c**) Glass fiber filter (placed in a tube after ablation). LS: nanosecond infrared laser system; TL1/2: telescope lens 1/2; M: mirror; FL: focusing lens; SM: scanning mirror; PC: computer; AC: ablation chamber with cooling element; AF: air filter array; MS: 3-axis manual displacement stage; S: frozen sample; FM: filter mount; GF: glass fiber filter; VP: vacuum pump; T: tube.

The ablation setup is depicted in Figure 7a. From the outlet of the pulsed nanosecond infrared laser system (Opolette SE 2731, Opotek, Carlsbad, CA, USA), the divergent beam passed through a telescope with two plano-convex lenses (ISP-PX-25-150 and ISP-PX-25-100, ISP Optics Latvia, Riga, Latvia) for collimation purposes, followed by a 150 mm focusing lens (ISP-PX-25-150, ISP Optics Latvia, Riga, Latvia) with a spot diameter of about 150 µm. The relatively long focal distance of 150 mm of the setup for focusing the beam formed a relatively long focal spot of about 2 mm in the axial direction with equal energy distribution. This elongated spot volume compensated for minor height deviations on the sample surface. A dual-axis scanning mirror (OIM202, Optics in Motion, Long Beach, CA, USA), controlled by a data acquisition input/output device (USB-6343, National Instruments, Austin, TX, USA), was used for transverse scanning. Laser triggering was synchronized to the scanning mirror and timed to match the maximum possible repetition rate of 20 Hz. The two-inch scanning mirror also allowed the integration of a camera path for aiming purposes. The tissue sample was placed on a cooling stage inside a closed ablation chamber with a glass window on the top and two tube connectors to establish an air stream. The inlet was equipped with an air filter to minimize contamination. The emerging tissue aerosol from the ablation was transported to the outlet by a membrane pump (Mz 2c Vario, Vacuubrand, Wertheim, Germany) [57], where it passed through a short steel tube before being trapped on a glass fiber filter with a diameter of approximately 10 mm (GF50 grade, glass fiber filter without binders, Hahnemühle FineArt, Dassel, Germany) that was placed in a stainless steel filter mount (Figure 7a,b). After each ablation, the filter was transferred to a tube (Figure 7c).

### 4.3. Laser Parameters and Tissue Sampling

The tunable wavelength (2.70–3.10 µm) of the NIRL was set to 2.94 µm to match the O-H vibrational stretching band of water. The pulse energy was set to 1500 µJ at the sample position. In the custom-made control software, the scanning area was set to a square of 1.6 mm × 1.6 mm containing a pattern of 15 × 15 laser shots with a lateral spacing of 114 µm for each layer. For each sample technical replicate, six layers were ablated, resulting in a total of 1350 applied laser shots and the removal of a volume of about 1750 µm × 1750 µm × 160 µm, which corresponded to about 500 nL. The volume was determined by reference measurements with optical coherence tomography, as in our previous studies, where we demonstrated that the NIRL ablation volume is reproducible for technical replicates in a specific tissue type [44,45]. During the ablation process, the frozen tissue sample was temperature controlled at −10 °C to maximize consistent tissue removal. After sampling, the used glass fiber filter was removed and stored in a tube at −24 °C. For this study, three ablations (technical replicates) were performed for each tissue sample. Before the next tissue sample was placed, the entire chamber and output tubing down to the filter mount were cleaned with isopropanol; the filter mount was also cleaned with isopropanol, as well as with an ultrasonic cleaner (USC100TH, VWR, Darmstadt, Germany) for 5 min.

### 4.4. Extraction of Lipids from the Tissue Aerosol Condensates

A Lipidyzer^TM^ Internal Standards Kit (AB SCIEX, Framingham, MA, USA) was prepared according to the manufacturer’s instructions and resuspended in methyl tert-butyl ether (MTBE). The glass fiber filter was mixed with 50 µL internal standard, 500 µL MTBE, and 160 µL methanol and then extracted for 30 min at 20 °C at a rotation speed of 400 rpm on a shaker (ThermoMixer® C, Eppendorf SE, Hamburg, Germany). After adding 200 µL water (LC-MS grade), the sample was centrifuged at 16,000× *g* for 5 min. The upper MTBE phase was removed and transferred to a new tube. After adding 300 µL MTBE, 100 µL methanol, and 100 µL water (LC-MS grade), the sample was mixed and centrifuged at 16,000× *g* (MIKRO 185, Andreas Hettich, Tuttlingen, Germany) for 5 min. The upper phase was removed and combined with the previous upper phase. The combined phases were dried in a vacuum concentrator centrifuge (UNIVAPO 100 H, UniEquip, Martinsried, Germany) and stored at −20 °C until further use. Prior to MS analyses, lipids were resuspended in 250 µL of 10 mM ammonium acetate in dichloromethane : methanol (50:50 (*v*/*v*)).

### 4.5. Lipidomic Analysis

Lipidomic analysis was carried out with a triple quadrupole mass spectrometer (QTRAP 5500; AB SCIEX, Framingham, MA, USA) equipped with a differential mobility spectrometer (DMS) interface operating with SelexION technology [58]. This device was coupled to an ultra-high pressure liquid chromatography system (Nexera X2, Shimadzu, Kyoto, Japan). The lipidomics platform (Lipidyzer^TM^) was operated with lipidomics algorithms (Analyst version 1.6.8 and Lipidomics workflow manager; AB SCIEX, Framingham, MA, USA). The Lipidyzer^TM^ Platform was tuned with a SelexION Tuning Kit (AB SCIEX, Framingham, MA, USA), and a system suitability test was performed with a System Suitability Kit (AB SCIEX, Framingham, MA, USA), both according to the manufacturer’s instructions. The Lipidyzer^TM^ Platform used 10 mM ammonium acetate in dichloromethane : methanol (50:50 (*v*/*v*)) as the running buffer, dichloromethane : methanol (50:50 (*v*/*v*)) as rinses 0 and 1, 2-propanol as rinses 2 and 3, and 1-propanol as a DMS modifier. 50 µL samples were injected for both multiple reaction monitoring (MRM) methods: one with DMS on and one with DMS off. A detailed description of this shotgun approach has been reported previously [59]. Data processing and quantification were performed automatically by the Lipidyzer^TM^ lipidomics workflow manager; lipid concentrations are given in nmol/mL.

### 4.6. Data Analysis and Visualization

Quantified lipid species concentrations were loaded into Perseus (version 1.6.15.0, Max Planck Institute for Biochemistry, Martinsried, Germany) and log2 transformed. Nonlinear iterative partial least squares (NIPALS) principal component analysis (PCA) was performed in RStudio (version 2022.07.1 + 554, Posit PBC, Boston, MA, USA), with 70% valid values in at least one patient. That corresponded to 13 lipid classes, 122 lipid species with the same fatty acyl chain length and extent of unsaturation, and 327 individual lipid species (Supplementary Table S2a–c). Results of the NIPALS PCA are visualized as scatter plots (Figure 5). Further testing was performed with Welch’s test. The results were filtered for *p*-value and fold-change significance (*p*-value ≤ 0.05, two-fold change). Significant lipid species are listed for each patient in Supplementary Table S3a–d.

## 5. Conclusions

In this pilot study, we have demonstrated that tissue sampling and homogenization utilizing a modified nanosecond infrared laser (NIRL) setup and consecutive mass spectrometric lipidome analysis is a promising approach for tissue classification and differentiation, which also has the potential to provide new insights into lipid composition alterations in OSPCC. To our knowledge, this study is the first to provide MS insights into the lipidome of this specific anatomic location. Subsequent studies with a larger patient population and with optimizations to reduce the minimum sample amount are necessary to fully explore the capacity of the NIRL MS platform and to prospectively enable in vivo tissue sampling with improved spatial resolution.

## Figures and Tables

**Table 1:** Demographic and clinical data for all patients.

Number of Patients (*n*)	4
Sex, (n (%))	
male	3 (75%)
female	1 (25%)
Mean age at surgery (years)	63
Age range (years)	51–69
OPSCC	
of the tonsils (n (%))	2 (50%)
of the base of the tongue (n (%))	2 (50%)

**Table 2:** Detailed demographic and clinical data for individual patients. BOT: base of the tongue; p16: p16 INK4A immunohistochemistry; HPV: human papilloma virus polymerase chain reaction for virus serotype 16.

Patient Number	Age	Gender	Tumor Location	Alcohol Consumption	Tobacco Consumption	OPSCC Samples per Patient	p16/HPV Status
A	69	M	BOT	Yes	No	2	+/+
B	64	F	BOT	No	Yes	2	+/+
C	66	M	Tonsil	Yes	No	1	+/+
D	51	M	Tonsil	Yes	Yes	2	+/+

**Table 3:** Origin of samples and correlation to patients. BOT: base of the tongue.

Patient	OPSCC Location	OPSCC Samples	Healthy Mucosa Samples
A	BOT	A-1	A-H
		A-2	
B	BOT	B-1	B-H
		B-2	
C	Tonsil	C-1	C-H
D	Tonsil	D-1	D-H
		D-2	

**Table 4:** Number of lipid species showing significant differences (*p*-value < 0.05, two-fold change) to lower or higher concentrations in OPSCC compared to the corresponding healthy tissue (Supplementary Table S3a–d).

	BOT				Tonsil		
	Patient A		Patient B		Patient C	Patient D	
Samples tested	A-1 vs. A-H	A-2 vs. A-H	B-1 vs. B-H	B-2 vs. B-H	C-1 vs. C-H	D-1 vs. D-H	D-2 vs. D-H
↓ in OPSCC	103	162	78	74	26	20	4
↑ in OPSCC	17	24	4	11	8	28	34

## Data Availability

Mass spectrometric raw data was automatically processed by the Lipidyzer Platform. The corresponding quantitative data can be found in Supplementary Table S1a–c.

## Supplementary Materials

The supporting information can be downloaded at: https://www.mdpi.com/article/10.3390/ijms24097820/s1.
